# Physical Frailty and Cognitive Impairment in Older Nursing Home Residents: A Latent Class Analysis

**DOI:** 10.1093/geroni/igab046.2999

**Published:** 2021-12-17

**Authors:** Yiyang Yuan, Kate Lapane, Jennifer Tjia, Jonggyu Baek, Shao-Hsien Liu, Christine Ulbricht

**Affiliations:** 1 University of Massachusetts Medical School, Burlington, Massachusetts, United States; 2 University of Massachusetts Medical School, University of Massachusetts Medical School, Massachusetts, United States

## Abstract

Physical frailty (PF) has various clinical presentations and often co-occurs with cognitive impairment in older adults. In older adults in nursing homes (NHs), no research has examined the heterogeneous profile of PF and its association with cognitive impairment. Minimum Data Set 3.0 was used to identify older, long-stay, newly-admitted NH residents (2014-16; n=871,801). Latent class analysis was used to identify PF subgroups with FRAIL-NH items as indicators. Logistic regression was used to estimate the association between PF subgroups and cognitive impairment. The final model indicated three PF subgroups (prevalence): “mild PF” (7.6%), “moderate PF” (44.5%), and “severe PF” (47.9%). In all subgroups, residents had high probability of needing help with dressing. Older adults likely to belong to the “moderate PF” or the “severe PF” subgroups had high probabilities of requiring physical assistance to transfer between locations and inability to walk in a room. Additionally, residents likely to be in the “severe PF” subgroup had greater probability of bowel incontinence. Greater cognitive impairment was associated with increasingly higher odds to be in the “moderate PF” and “severe PF” subgroups: older residents with severe cognitive impairment were 20% more likely [adjusted odds ratio (aOR): 1.20, 95% confidence interval (CI): 1.17-1.23] and almost 7 times as likely (aOR: 6.86, 95%CI: 6.66-7.06) to belong to the “moderate PF” and “severe PF” subgroups, respectively. Findings provide new evidence for the interrelationship between PF and cognitive impairment in older NH residents and have implications for the development of interventions tailored to older residents’ specific PF experience.

